# Barriers and Strategies for Oral Peptide and Protein Therapeutics Delivery: Update on Clinical Advances

**DOI:** 10.3390/pharmaceutics17040397

**Published:** 2025-03-21

**Authors:** Kshitis Chandra Baral, Ki Young Choi

**Affiliations:** 1 Department of Marine Bio-Food Science, Gangneung-Wonju National University, Gangneung 25457, Republic of Korea; r1189@gwnu.ac.kr; 2 NVience Inc., Seoul 04323, Republic of Korea

**Keywords:** protein therapeutics, enzymatic degradation, oral drug delivery, bioavailability enhancement

## Abstract

Peptide and protein (PP) therapeutics are highly specific and potent biomolecules that treat chronic and complex diseases. However, their oral delivery is significantly hindered by enzymatic degradation, instability, and poor permeability through the gastrointestinal (GI) epithelium, resulting in low bioavailability. Various strategies have emerged as transformative solutions to address existing challenges, offering enhanced protection, stabilization, and absorption of PPs. These strategies primarily focus on two major challenges: protecting the PP against harsh conditions and enhancing permeation across the intestinal membrane. Innovative approaches such as pH modulation and incorporation of enzyme inhibitors are usually used to mitigate proteolytic degradation of PP during transit across the GI tract. In a similar vein, absorption enhancers and prodrug strategies facilitate epithelial transport, while targeted delivery systems focus on specific areas of the GI tract to enhance absorption. Likewise, mucus-penetrating and mucoadhesive strategies have enhanced retention and interaction with epithelial cells, effectively overcoming barriers like the mucus layer and tight epithelial junctions. Furthermore, structural modifications such as lipidation, peptide cyclization, and polyethylene glycosylation are promising alternatives to render stability, prolong circulation time, and membrane permeability. In particular, functional biomaterials, active targeting, and lymphatic transport strategies have provided new platforms for oral PP delivery. Advancing in materials science, nanotechnology, and the disruption of medical devices holds new frontiers to overcome barriers. Despite substantial advancements, the limited success in clinical translation underscores the urgency of innovative strategies. This review presents oral PPs as a promising platform, highlighting the key barriers and strategies to transform their therapeutic landscapes.

## 1. Introduction

Recent advancements in biotechnology and molecular biology have led to the development of new biotherapeutics based on peptides and proteins. These peptide and protein (PP) therapeutics offer unique biological functions and are generally well-tolerated upon administration. The global PP therapeutics market has been on a robust growth trajectory. It was valued at $42.8 billion in 2023 and is expected to grow at a compound annual growth rate of 7.9% between 2024 and 2032, potentially surpassing $80 billion by 2033 [1]. This growth aligns with the increasing share of biologics in new drug approvals, which has consistently ranged between 30% and 40% in recent years. Thus, almost half of the new molecule entities currently being developed by pharmaceutical companies are PP therapeutics [2].

These drugs possess significant therapeutic potential owing to their high specificity, potency, and ability to target diverse biological pathways [3]. Recently, they have been used to treat various conditions such as diabetes, cancer, cardiovascular diseases, inflammatory disorders, and hormonal imbalances [4]. Several clinical trials of PP therapeutics have been initiated, and many products have been approved—including salmon calcitonin, octreotide, and glucagon-like peptide 1 (GLP-1) agonists (e.g., semaglutide)—to improve the oral delivery of peptide therapeutics [5,6,7]. The growth of PP therapeutics has been driven by the rising prevalence of chronic diseases, advances in biotechnology, and their unique target specificity. Owing to their high specificity in binding capacity with their targets in vivo, PPs offer higher potency and specificity than small-molecule drugs, often resulting in more pronounced effects with fewer adverse side effects [8].

Most of these PPs are currently limited to intravenous injections, owing to their inherent short in vivo biological half-lives due to their rapid clearance in the liver and other body tissues by proteolytic enzymes [9]. However, parenteral administration burdens patients because of its invasive nature, leading to risks of immunogenic effects and poor compliance [10]. Notably, the requirement of administration by injection is associated with low patient compliance, especially in chronic treatments. Therefore, finding an alternative and non-invasive route to administer PP therapeutics has become a focal point of biopharmaceutical research.

Oral administration is the preferred and most convenient route of drug administration, as it is associated with higher patient compliance, lower risk of immunogenicity, and lower production costs compared to injectables. Despite the therapeutic potential of PPs, oral administration is often limited by inherent biopharmaceutical limitations such as limited solubility in the intestinal environment, short half-life, high molecular weight, hydrophilicity, susceptibility to enzymatic degradation, low permeability across the intestinal epithelium, and low stability in the gastrointestinal (GI) tract—all of which ultimately lead to poor oral bioavailability [11,12,13]. These key barriers demand extensive research on developing innovative strategies to improve the oral stability, absorption, and bioavailability of PP therapeutics.

Recent advancements have drawn attention to developing various oral PP delivery systems. These systems are usually integrated with functional excipients, including pH modulators, enzyme inhibitors, absorption enhancers, cell-penetrating peptides, hydrophobic ion pairing (HIP), and mucoadhesive polymers. Various drug delivery systems (DDSs)—including microemulsions, self-emulsifying DDSs (SEDDS), liposomes, solid lipid nanoparticles (SLNs), nanostructured lipid carriers (NLCs), lipid-polymeric hybrid systems (LPHs), hydrogels, and other smart-ingestible medical device have shown promising results in terms of overcoming the barriers to PP delivery [14,15,16]. Such strategies extend residence time, target specificity, and controlled release kinetics, substantially improving the oral bioavailability of PPs.

Despite this significant progress, specific challenges persist in designing reproducible oral delivery systems. The intricate interactions of factors such as drug release kinetics, particle size, carrier stability, and the impact of food intake necessitate continuous optimization and improvement. Numerous reviews on oral PPs emphasize theoretical advancements or preclinical findings, often overlooking clinical translation. This review outlines the challenges to delivering PPs orally and highlights various innovative research strategies used to address them, with a primary focus on clinical applications. We also assess emerging smart-ingestible device technologies aiding oral PP delivery, a rarely explored topic, and evaluate their effectiveness in overcoming physiological barriers. Furthermore, we examine ongoing clinical trials, new product approvals, and regulatory issues, providing a comprehensive overview of the evolving oral PP therapeutics landscape. By focusing on translational impact rather than just experimental progress, this review offers an organized perspective that transcends traditional discussions.

## 2. Challenges to the Oral Delivery of Peptide and Protein Therapeutics

The oral route is the most convenient method for administering PP-based drugs. It is advantageous because of its noninvasiveness, ease of administration, and general acceptability to patients [17]. PP therapeutics are commonly classified as biopharmaceutical classification systems (BCS) III (low permeability, high solubility) or IV (low permeability, low solubility) [18], with typical oral bioavailability levels of <1% and sometimes even <0.1% [10,19]. The primary barriers to PP absorption through the GI tract include chemical, enzymatic, and penetration-related factors, such as the gastric mucosal and intestinal epithelium layers [20]. The critical challenges are (i) protecting these compounds from enzymatic degradation in the harsh GI environment, (ii) preventing systemic elimination, and eventually (iii) improving the oral absorption of PPs [21].

### 2.1. Physicochemical Properties

The intrinsic properties of PPs, including their large molecular size, hydrophilicity, limited membrane permeability, and susceptibility to enzymatic degradation, pose challenges for oral delivery systems [22,23]. These properties affect encapsulation efficiency, retention, absorption, permeation, stability, and release rate [24,25]. Because of their complex secondary, tertiary, and quaternary structures, PPs can also lose bioactivity via interactions with various environments during manufacturing or storage through mechanisms such as denaturation, adsorption, aggregation, oxidation, and hydrolysis [26]. Factors such as pH, temperature, agitation, ionic strength, and the presence of metal ions or surfactants can also significantly impact their stability [26].

The high molecular weights and large sizes of PPs often lead to poor membrane permeability and low absorption. Drugs with molecular masses < 500 Da can generally pass through GI membranes via passive diffusion; however, proteins ranging between 1 and 100 kDa encounter limited membrane penetration because of their larger sizes [27,28]. The high hydrophilicity of PP therapeutics, which generally have log(*p*) values < 0, significantly affects their ability to cross cell membranes, as epithelial cell membranes must break their hydrogen bonds with water to interact with the lipid bilayers of cellular membranes [29,30]. Passive absorption in the GI tract is mainly determined by molecular size, as the organized and densely-packed lipid bilayer structures of epithelial cell membranes limit the entry of large biomolecules. Moreover, the lipophilic nature of the biological membranes and the narrow paracellular space (3–10 Å) further restricts diffusion through paracellular pathways [28]. As a result, passive transcellular uptake is significantly restricted. Small molecules can passively diffuse down their concentration gradients [30], but not the PPs, whose cellular uptake is primarily governed by active transport or endocytosis rather than passive diffusion [18].

A significant drawback of the endocytic pathway is the possibility of endosome entrapment, which often leads to lysosomal degradation [31]. A key physicochemical property affecting the absorption of oral PPs is surface charge, which depends on the protein’s amino acid composition and the pH of the surrounding environment. The impact of surface charge on passive drug diffusion across the GI tract has been well documented, with the tract’s epithelium generally being less permeable to charged molecules over their uncharged counterparts because of their lack of lipophilicity [30]. Surface charge can govern interactions between PPs and cell surface molecules, thus affecting absorption, distribution, and elimination.

The solubility of PPs is highly pH-dependent, being at its lowest at each PP’s isoelectric point (pI). At this point, PPs exist as zwitterions, which have a negative effect on membrane permeability [32]. When predicting oral permeability, the electrostatic charge of a PP may be more significant than its partition coefficient. Changes in surrounding pH alter the charges and ionization states of PPs, thus affecting their abilities to cross cellular membranes. At physiological pH levels or above the pI, epithelial proteins carry negative charges that favor the binding of positively charged drugs and vice-versa [30].

Likewise, oral delivery of PP therapeutics is susceptible to destabilization by various proteolytic enzymes and physicochemical factors, which can occur during manufacturing and systemic exposure [33]. These destabilization phenomena are complex and heterogeneous, often resulting from deamination, isomerization, or post-translational modifications that can alter net charges and lead to the formation of acidic and basic variants [34]. Understanding the physicochemical properties of PPs is essential to creating effective oral formulations.

### 2.2. Biological Barriers

#### 2.2.1. Luminal Barrier (pH)

**Figure 1:**
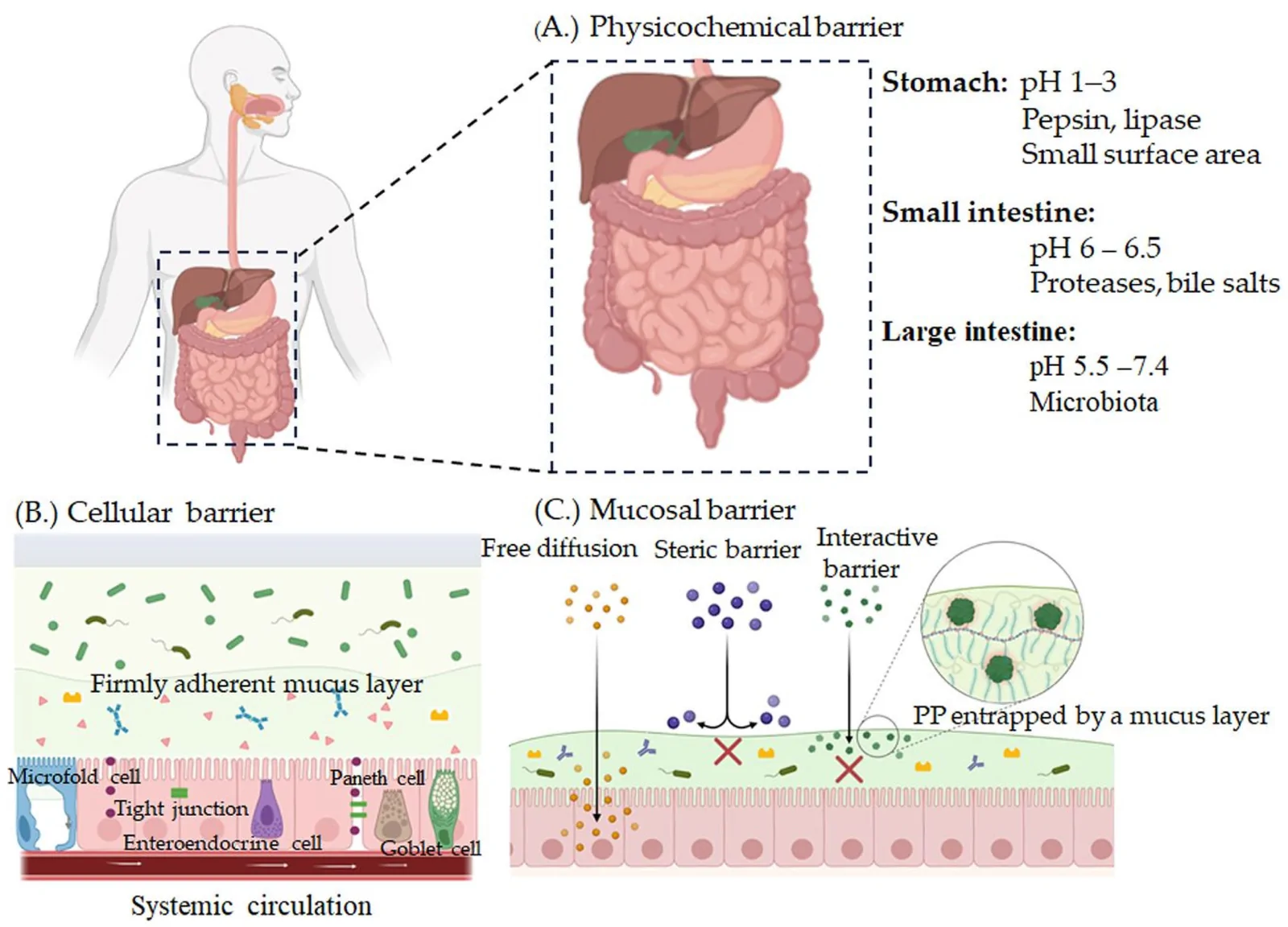
Barriers to oral PP delivery. (**A**) Physicochemical barrier. The harsh conditions of the GI tract, including pH, enzymatic degradation (such as pepsin, lipase, proteases, and bile salts), and microbial activity, compromise the stability and absorption of oral PPs. (**B**) Cellular barrier. The intestinal epithelium contain specialized cells such as microfold cells, goblet cells, paneth cells, and enteroendocrine cells as well as tight junctions, which restrict paracellular transport of PPs into systemic circulation. (**C**) Mucus barrier. The mucus layer serves as both a steric and interactive barrier, restricting PP diffusion and leading to entrapment, which reduces its overall bioavailability. The figure was created using Biorender.com.

PP therapeutics encounter significant challenges when administered orally, owing to the varying pH levels throughout the GI tract that can lead to degradation and reduced biological activity [10] (Figure 1A). The gradient of pH conditions they encounter in different biological environments can influence the ionization, chemical stability, and absorption of oral PP preparations. Upon encountering a highly acidic environment in the stomach (pH 1.0–2.0), PPs become susceptible to denaturation, unfolding, and inactivation in the presence of proteolytic enzymes. The pH then gradually increases through the duodenum (pH 4.0–5.5), jejunum (pH 5.5–7.0), and ileum (pH 7.0–7.5) [10]. The chemical degradation of PPs decreases correspondingly—particularly in the ileum and colon, where pH levels range from 6 to >8 [29]. PPs are only stable over narrow pH ranges near their isoelectric point (pIs). When pH levels are above or below this point, PPs carry negative or positive charges, respectively. These charges make PPs more hydrophilic, reducing their ability to cross neutral cellular membranes [35,36].

Enzymatic activity in the GI tract is highly dependent on pH. For example, the stomach enzyme pepsin works best in an acidic environment (pH 2–3), which can lead to the rapid degradation of certain PPs. However, pepsin becomes inactive at pH levels > 5 [35]. On the other hand, the enzymes of the small intestine, such as trypsin and chymotrypsin, function optimally at higher pH levels. The situation is further complicated by factors including the presence of food, pathological conditions such as inflammatory bowel disease and GI cancers, as well as individual differences in diet [35]. These factors can influence the pH environment, leading to more significant variability and challenges to the stability and absorption of PPs [35]. Therefore, effective oral delivery systems must consider pH-based factors to ensure the protection and stability of PP therapeutics throughout the GI tract.

#### 2.2.2. Enzymatic Barrier

There are many enzymatic barriers to the oral delivery of PPs, involving various digestive enzymes throughout the GI tract. Orally administered PPs are highly susceptible to digestion by proteolytic enzymes, particularly in the colon. These include luminal enzymes, pancreatic secretions, and mucosal and bacterial enzymes (Figure 1A) [37]. These biochemical barriers significantly hinder the uptake of PPs, leading to decreased bioavailability. In the stomach, gastric glands secrete digestive fluids containing hydrochloric acid, pepsin, and mucus. Under a highly acidic environment (pH 1–2), pepsin functions optimally as a broad endopeptidase that hydrolyzes the peptide bonds of proteins with aromatic residues (phenylalanine, tryptophan, and tyrosine) into smaller peptides to expand their accessibility to pancreatic proteases [13,38]. The epithelial cell border also presents an enzymatic barrier, with ~15 enzymes that target various macromolecules. Peptidases in the GI tract rapidly degrade PPs [39]. Consequently, their absorption is significantly lower in the stomach than in the intestines [40]. Significant gastric absorption occurs only for PPs that are stable at low pHs and lack pepsin cleavage sites. In addition, lipase enzymes catalyze the breakdown of fats, oils, and triglycerides in the stomach. Digestion further continues in the small intestine, which is rich in several pancreatic enzymes. The serine proteases trypsin, α-chymotrypsin, elastase, endopeptidases, and the exopeptidases carboxypeptidases A and B lead to the rapid degradation of most PPs in this organ [41,42]. Proteolytic enzymes in the GI tract—including pepsin, trypsin, elastase, chymotrypsin, and carboxypeptidase—require an optimal acidic environment. For instance, under physiological conditions, insulin is nearly completely degraded by trypsin, α-chymotrypsin, and elastase within 1 h [43].

Ingested PPs may also be degraded in the intestinal lumen as well as by membrane-bound enzymes on the brush border membrane. Brush border enzymes are glycoproteins that can be subdivided into endopeptidases, N- and C-terminal slicing exopeptidases, and dipeptidases [44]. In addition to lumen-secreted and brush border membrane-bound enzymes, cytosolic enzymes in enterocytes such as lysozymes play a role in this enzymatic degradation process [45]. Lysozyme enzymes are essential for the uptake of lipophilic PPs through transcellular pathways. In the colon, enzymes produced by local microflora further contribute to the degradation of orally ingested PPs, breaking them down into byproducts such as short peptide chains and amino acids that are typically ineffective in terms of providing the desired therapeutic effect. Understanding these processes is crucial to the development of strategies to enhance the bioavailability of orally delivered PPs.

#### 2.2.3. Epithelial Barrier

The highly vascularized intestinal mucosa requires drugs to traverse only a single layer of epithelial cells before they reach the bloodstream and systemic circulation. However, its regulation presents a significant challenge to the oral delivery of PPs. The epithelial layer mainly comprises enterocytes separated by tight junctions, which are composed of four unique transmembrane proteins: occludin, claudins, junctional adhesion molecules, and tricellulin (Figure 1B) [13,35]. These junctions are dynamic structures that regulate the penetration of molecules across the intestinal epithelium.

Compounds such as drugs can enter the bloodstream from the intestinal lumen through various pathways: transcellular, paracellular, endocytosis/transcytosis, or carrier-mediated transport [43]. The passage of PPs across this cell layer may be hindered for two reasons: those with hydrophilic natures cannot undergo transcellular permeation across the cells’ lipophilic plasma membranes, and those that are too large cannot exploit the paracellular pathway through the small gaps of tight junctions [46]. Consequently, larger hydrophilic PPs generally cannot follow the transcellular route of absorption via passive diffusion [9]. Notably, the absorption of PPs is limited by the rapid decrease in membrane permeability for molecules of >1 kDa size [43].

Hydrophilic PPs cannot partition into the lipid bilayers of epithelial cells, which further restricts their entry [43]. The paracellular route represents an aqueous extracellular alternative that may be viable for PP delivery, owing to a possible scarcity in the abundance of proteolytic enzymes [47]. Paracellular spaces constitute < 1% of the total mucosal surface, and tight junctions between epithelial cells must be transiently opened to facilitate systemic uptake [43,48]. It has been demonstrated that the paracellular route is not viable for PP absorption because these large molecules cannot generally fit into these spaces. As a result, most PPs cannot traverse this barrier without the aid of auxiliary agents and novel formulation strategies. A number of studies have recently explored the properties of the epithelial barrier and various ways to bypass it, highlighting the necessity for innovative approaches to enhance their absorption and bioavailability. Penetration enhancers, for example, can temporarily loosen tight junctions and thus facilitate the paracellular absorption of PP therapeutics.

#### 2.2.4. Mucus Barrier

The GI tract has two distinct mucus layers: firmly and loosely adherent [35]. The firmly adherent layer is directly adjacent to the epithelial lining and consists of the glycocalyx’s cell-bound mucins, glycolipids, and glycoproteins [49]. By contrast, the loosely adherent mucus layer undergoes constant turnover, with a dynamic behavior characterized by continuous secretion and sloughing-off from the mucosal membrane surface, creating a formidable gel barrier (Figure 1C) [10]. The mucus layer of the intestinal lumen functions as both a physical and interactive barrier, significantly impeding the permeation of pathogens, toxins, and large molecules (including PPs) through and within the mucus [49,50].

The mucus layer is a viscoelastic, hydrogel-like substance secreted by goblet cells that line the GI tract and are linked via disulfide bonds that create a mesh-like structure with shear-thinning properties [51]. These rigid structures are composed of mucins, ions, and glycoproteins arranged in a dense three-dimensional network, with variations in thickness and turnover depending on anatomical location and pathophysiological conditions [10]. It also contains water, carbohydrates, lipids, electrolytes, immunoglobulins, antimicrobial peptides, protease inhibitors, active proteins, bacteria, and other cellular debris [13,51]. Mucins, the predominant component of mucus, are a heavily glycosylated class of glycoproteins with a charged, bottlebrush-like structure that promotes gel formation [10,13].

This barrier limits drug permeation via two mechanisms: binding to drugs with negatively charged mucin fibers or physical hindrance to permeation through the mesh structure [52]. Various interactions within the mucus, including ionic interactions, hydrogen bonding, and hydrophobic interactions, collectively create a significant barrier to PP diffusion (Figure 1C) [49,53]. For instance, the negative charge of mucus, which is primarily attributed to sialic and sulfonic substructures, impedes the permeation of cationic peptides through ionic interactions [54]. PPs must first traverse through a 100–200-µm thick mucus gel layer to reach the lymphatic membrane that covers the GI epithelium [15]. The average pore size of the mucus is ~0.2 μm, though this can vary based on location and responses to endogenous and exogenous stimuli, potentially allowing PPs to penetrate [55,56]. Studies have indicated that peptides of >6.5 kDa size can permeate this mucus gel layer to a limited extent, while the rate of permeation is almost negligible for those with molecular masses > 12.4 kDa [57]. This indicates that the mucus gel layer acts as a selective barrier, allowing only smaller or modified peptides to reach the underlying epithelial cells and be absorbed.

These layers are also crucial for regulating the pH at the surface of the GI lumen. The pH of mucus varies depending on its location. For instance, the gastric mucus layer exhibits a pH gradient, with the luminal surface being highly acidic (pH 2.25) compared to areas near the epithelial interface (pH 6.96) [10]. As a result, the pI of a PP significantly affects its solubility in the GI tract and ability to diffuse through the mucus. Understanding these limitations is essential for developing effective strategies to enhance the delivery and absorption of peptide drugs, such as designing smaller peptides, modifying peptide structures to reduce interactions with the mucus, or using delivery systems that can transiently disrupt the mucus barrier.

#### 2.2.5. Sulfhydryl Barrier

PP therapeutics with thiol or disulfide structures are susceptible to thiol/disulfide exchange reactions in the GI tract [58]. This interaction often leads to the formation of inactive conjugates. In addition to endogenous thiols such as glutathione and mucus glycoproteins with cysteine-rich subdomains, dietary proteins are involved in this process [43]. When administered orally, a sulfhydryl barrier affects PPs with disulfide groups, leading to a thiol-disulfide reaction. Thiols are primarily derived from food sources such as vegetables and fruits that contain glutathione, N-acetyl cysteine, homocysteine, cysteine, and γ-glutamyl cysteine. These antioxidants can potentially inactivate oral PPs.

## 3. Strategies for Improving Oral PP Absorption

The majority of oral PPs have extremely low bioavailability (often less than 0.1%), even with the use of various strategies. For the clinical advancement of oral PPs, a multifaceted approach that integrates peptide engineering and the selection of an innovative drug delivery system is crucial for addressing existing challenges. During the delivery system design, one critical factor is selecting highly potent peptides so that even small absorbed amounts can exert a meaningful pharmacological effect. For instance, oral semaglutide (Rybelsus^®^), an FDA-approved GLP-1 receptor agonist, has about 1% bioavailability; however, its high potency allows it to remain clinically effective [59]. Likewise, selecting peptides with prolonged half-lives also minimizes dose-to-dose variability, ensuring stable plasma levels, reducing dosing frequency, and enhancing therapeutic effectiveness and patient compliance.

**Figure 2:**
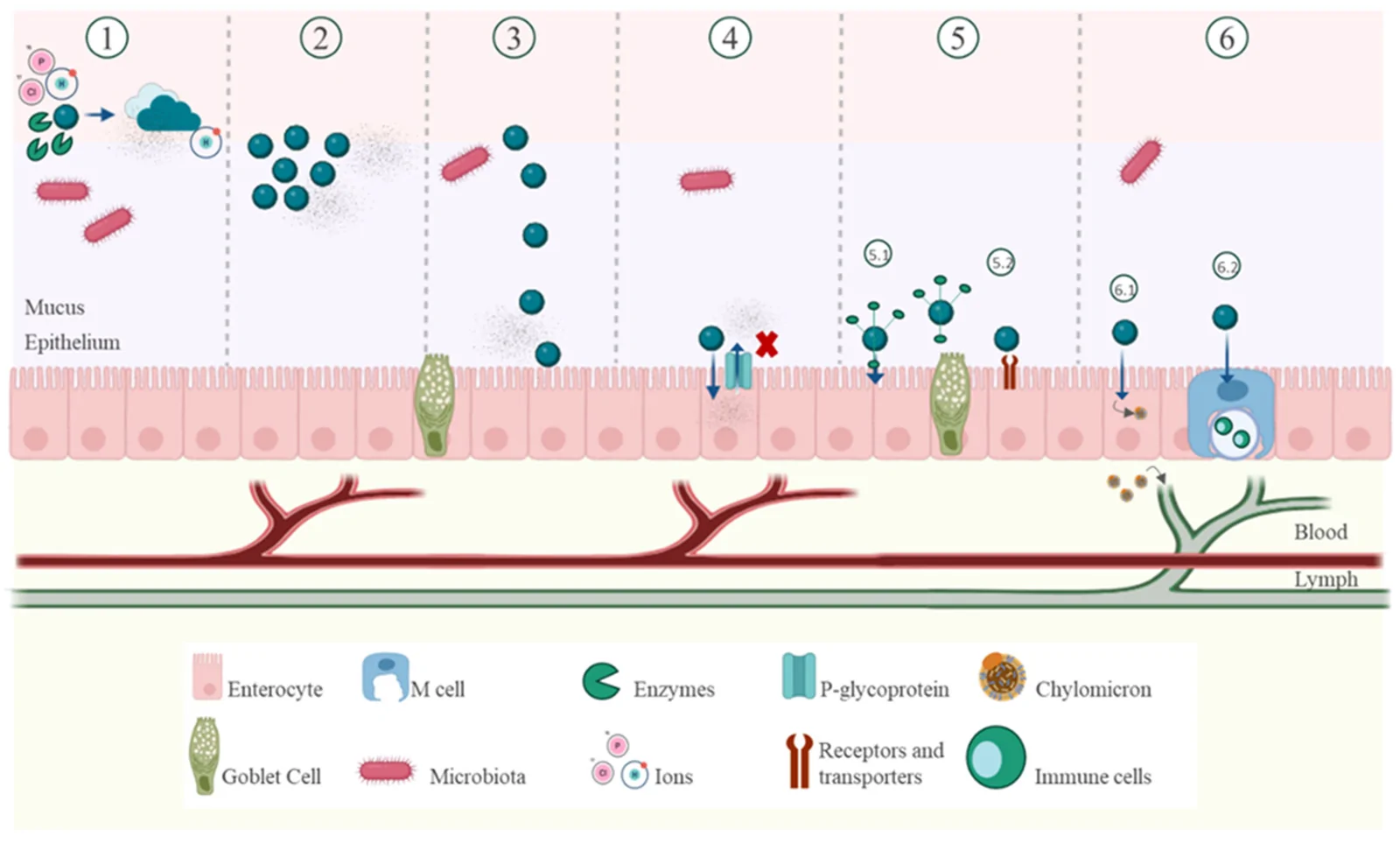
Various strategies for efficient oral delivery of PPs (1) Stabilization of PPs in the harsh gastrointestinal environment against enzymes, salts, and microbiota. (2) Mucoadhesive system. (3) Enhancement of mucodiffusion through the mucus-penetrating agents. (4) Inhibition of drug efflux mechanism. (5) Active targeting. (5.1) Functionalized ligands that interact with specific cell populations (enterocytes or goblet cells). (5.2) Delivery system act as targeting ligands by themselves. (6) Enhancing lymphatic transport system. (6.1) Chylomicrons, including lipids and hydrophobic cargo molecules from the internalized nanocarriers, are generated within enterocytes and absorbed by the lymphatic system. (6.2) Lymphatic uptake can also be achieved via M cells. The figure was created using Biorender.com. Reproduced with permission from [62], Springer Nature, 2021.

Several strategies for enhancing the proteolytic stability and membrane permeability of oral PPs include modifying the amino acid sequence by incorporating non-natural amino acids, cyclization, D-amino acid substitution, N-acylation, PEGylation, and glycosylation [60]. In addition to peptide design, formulation strategies such as the inclusion of mucoadhesive systems, penetration enhancers, and enteric coatings are essential to protect PPs from gastric degradation and enable targeted delivery to the intestine, improving localized absorption or therapeutic action. More recently, Nimble Therapeutics (recently owned by AbbVie) has utilized advanced peptide discovery platforms, including high-throughput screening, stabilization strategies, and the development of peptide conjugates with improved bioavailability. Various novel oral peptides such as C5 inhibitor (generalized myasthenia gravis), IL-23R inhibitor (psoriasis), and TL1A inhibitor (inflammatory bowel disease, phase 2) have been under clinical investigation [61]. Despite the existence of multiple strategies to enhance the oral absorption of PPs, the main principles focus on three key areas: stabilization, mucus penetration or adhesion, and permeation enhancement (Figure 2). These approaches are often integrated into single delivery systems. To date, several strategies have been developed to enhance the oral bioavailability of PPs—including the use of absorption enhancers and enzyme inhibitors in the formulations, development of mucoadhesive polymeric and particulate delivery systems, structural modifications of macromolecules, site-specific delivery, and use of cell-penetrating peptides (Table 1).

### 3.1. Stabilization

#### 3.1.1. pH Modulation

GI tract enzymes are primarily responsible for the degradation of orally administered PPs, but their activity requires optimal pH environments in order for them to exert their effects. For instance, pepsin can readily cleave multiple PPs in acidic environments; however, its activity diminishes when the pH rises to >3 [63]. Similarly, pancreatic enzymes in the small intestine, such as trypsin and chymotrypsin, are highly effective for degrading PPs, with optimal activity levels at pH levels ≥ 6.5 [64]. Thus, modifying the pH microenvironment may protect PPs from degradation. Strategies such as enteric coatings, rather than pH modulation, are often used to protect PPs from degradation in the stomach, as exploiting formulation is typically easier than employing pH modulators.

Once such PPs with enteric coatings reach the intestines, the higher pH dissolves the coating and releases the drug [65,66]. Adjusting the intestinal pH has also proven effective for protecting PPs. Some organic acids, such as citric acid, have been used as pH-lowering agents to inhibit the activity levels of intestinal enzymes [67].

#### 3.1.2. Enzyme Inhibitors

Enzyme inhibitors are promising strategies for counteracting the activity of intestinal enzymes. Proteolytic enzyme inhibitors deactivate target enzymes by binding to their specific sites, either reversibly or irreversibly [35,68]. Various enzyme inhibitors—including amino acids and their derivatives, peptides and their derivatives, and polypeptide protease inhibitors—have been incorporated into oral PP delivery systems [18]. Chemical compounds such as cholic acids and their derivatives, diisopropyl fluorophosphate, camostat mesylate, and *p*-amino benzamidine inhibit enzyme activity. However, owing to their high toxicity levels, these chemicals are rarely used. Their low molecular masses imply that they are absorbed faster than PPs, leading to systemic side effects and loss of inhibition capacity. Amino acids and their modified counterparts also experience problems similar to those of chemical inhibitors [69]. The development of enzyme inhibitors from peptides and modified peptides has been the subject of considerable research.

Proteolytic enzyme inhibitors such as aprotinin (inhibitor of trypsin and chymotrypsin), leupeptin (inhibitor of plasmin, trypsin, papain), soybean trypsin inhibitor, chicken ovomucoid (trypsin inhibitor), and FK448 (chymotrypsin inhibitor) can potentially enhance the absorption of PPs through the intestinal walls [11]. However, the prolonged use of such enzymatic inhibitors may lead to unpredictable interactions with dietary proteins, increasing pancreatic protease secretion and potentially causing enzyme deficiencies.

#### 3.1.3. Peptide Cyclization and Polyethylene Glycosylation

Cyclization is a chemical method used in peptide delivery to enhance stability by removing exposed N- and C-termini, which are particularly susceptible to enzymatic degradation [70]. It is typically achieved by establishing chemical bridges between different functional groups within the peptide. These bridges include disulfide bonds, lanthionine, dicarba, hydrazine, or lactam linkages between side chains [30]. There are four main approaches for cyclization: head-to-tail (connecting the N-terminus to the C-terminus), head-to-side chain, side chain-to-tail, and side chain-to-side chain [71]. These rigidify the PP structures, providing resistance to proteolytic enzymes and decreasing intermolecular hydrogen bond formation. Cyclization offers several advantages for PPs, including enhanced peptide stability against proteolytic enzymes, improved bioavailability through reduced hydrophilicity, and prevention of chemical degradation by rigidifying their structures. However, it has certain limitations, such as the complexity of the synthesis process, owing to the need for specific bridges and the potential loss of biological activity if the bioactive conformation of the PP is compromised. Despite these drawbacks, cyclization remains a promising strategy for improving the stability and oral bioavailability of PP drugs.

Similarly, polyethylene glycol (PEG) can be covalently attached to PPs through a form of glycosylation known as PEGylation, primarily to enhance their half-lives by creating steric hindrance that protects against degradation by proteolytic enzymes [72]. This approach is often used to reduce the plasma clearance rate of PPs, thereby improving their stability in the systemic circulation. While not all PPs are amenable to cyclization, direct PEGylation is a widely used alternative involving the covalent conjugation of peptide drugs with PEG.

The primary benefits of PEGylation include protection against proteases and improved intestinal permeability. It can also enhance their pH and thermal stability, as well as their resistance to proteolytic degradation in the GI tract [73,74]. However, it also increases the molecular size, leading to increased viscosity, reduced cell affinity, and limited biological activity [75]. Its higher molecular mass can enhance the pharmacokinetic and pharmacodynamic properties of PPs [76]. However, the potential drawbacks of the non-biodegradable nature of PEG can lead to adverse effects, such as changes in the drug’s efficacy and side effects compared to those of the original molecule, which must also be considered.

#### 3.1.4. Lipidation

Lipidation is a post-translational modification strategy used to enhance the stability and oral bioavailability of PPs [77]. The involvement of a lipid group in a peptide drug preserves its ability to bind to target receptors while modulating their hydrophilicity, secondary structures, and propensities of self-assembly. Increasing the lipid character of hydrophilic PPs by lipidation enhances mucosal permeability and metabolic stability, resulting in higher oral bioavailability than that of unmodified PPs [78]. Lipidation methods are categorized into covalent and non-covalent. Covalent lipidation, including PEGylation, acylation with various chain lengths of fatty acids, and polymer conjugation, can enhance lipophilicity by covalently conjugating lipophilic molecules to the target. These methods offer stable modifications and can deliver targets through ligand conjugation. However, they can cause changes in the secondary structure, thus potentially affecting the target receptor’s binding affinity, self-assembling property, biological activity, and pharmacokinetic profile [78]. Moreover, the product of covalent lipidation is considered a new drug entity distinct from the parent compound and thus likely requires separate approval [79]. Long fatty acid chains often enhance stability and systemic absorption across intestinal membranes by increasing lipophilicity, thus protecting against enzymatic degradation. Lipid attachment can occur through stable or labile linkages, forming a pro-drug. For instance, palmitoylation enhances the lipophilicity of insulin by attaching 1,3-dipalmitoylglycerol through an ester bond, improving intestinal penetration and stability against enzymatic degradation [80]. However, a significant challenge when using lipidized PPs for oral delivery is the lack of systematic studies on these enhanced oral absorption mechanisms. Several factors, including the linker used in lipid conjugation, the type of nanocarrier, and the presence of other excipients, can affect the oral bioavailability of a lipidized peptide.

**Figure 3:**
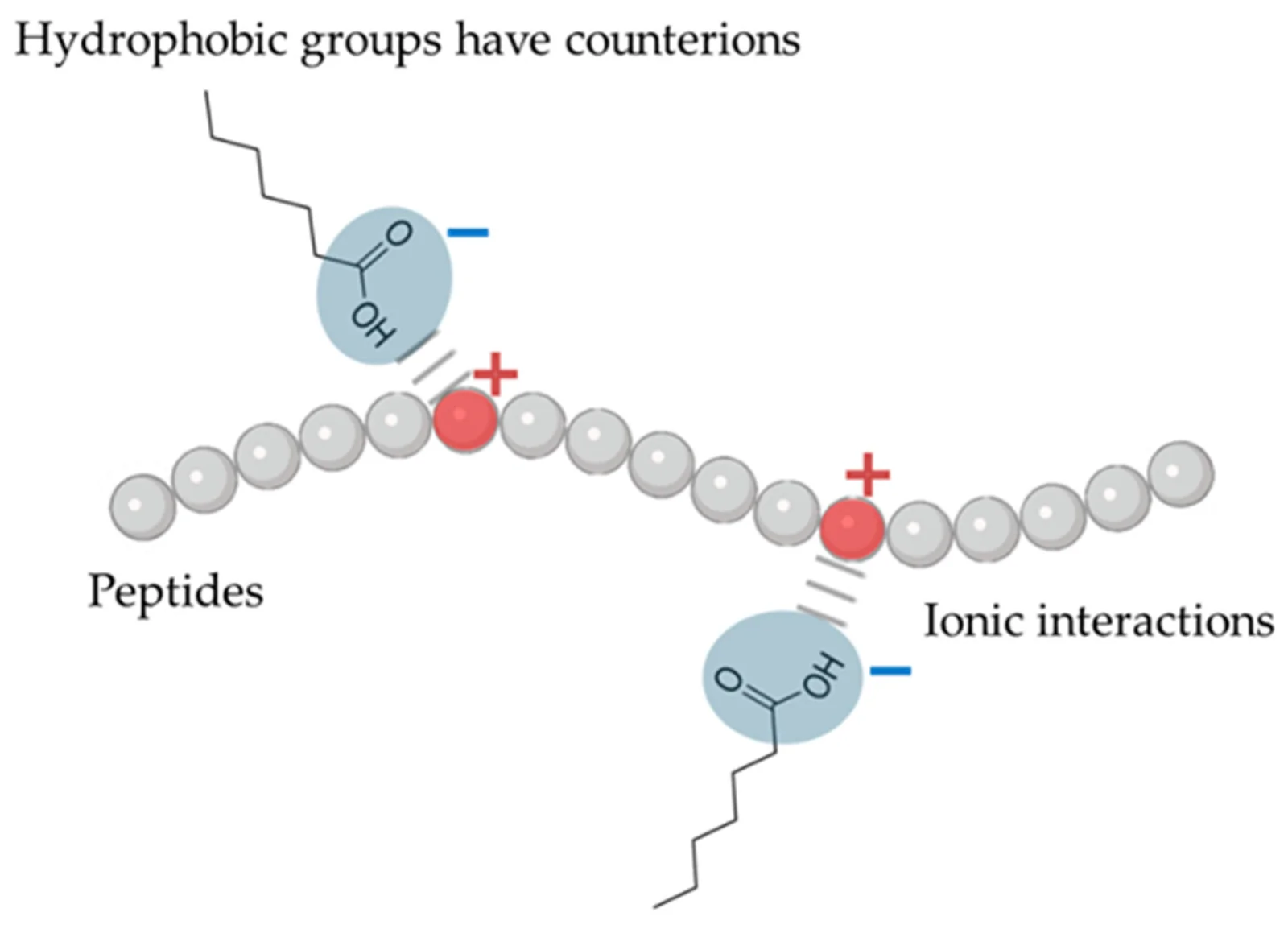
Hydrophobic ion complex (HIP) of peptide molecules with counterions via ionic interactions. The figure was created using Biorender.com.

In addition to chemical modifications such as covalently attaching fatty acids to peptides via ester or amide bonds, effective non-covalent lipidation strategies exist, including reversible aqueous lipidation, cyclization, hydrogen bonding, ionic interactions, complexation with divalent metal ions, and HIP. These methods are particularly relevant for the oral delivery of PPs. These reversible, non-covalent interaction-bound moieties can dissociate during absorption, restoring the original structure and, thus, leaving target binding affinity unaffected. They do not modify any covalent bonds of the original active pharmaceutical ingredients (API), and approval of the end product is not strictly required. However, a molecular-based mechanistic understanding remains incomplete [81]. Among the various methods, ionic interaction is the most effective for achieving non-covalent lipidation of oral PPs. Because most PPs contain at least one ionizable amino acid, their lipophilicity can be enhanced by neutralizing them with counterions that have at least one hydrophobic domain [82]. HIP involves coupling a charged hydrophilic molecule with an oppositely charged hydrophobic counterion, resulting in a more hydrophobic complex than the original molecule (Figure 3) [79]. This non-covalent lipidation technique increases lipophilicity and membrane permeability through physical complexation without causing significant or irreversible changes to secondary or tertiary structures. More specifically, it is based on an ionic interaction between the charged hydrophilic molecule and a counterion with at least one hydrophobic domain and an ionizable functional group in an aqueous medium. Various ionic surfactants (bile salts, sodium dodecyl sulfate, sodium docusate, and others) are commonly used as counterions for HIP, as they have both a hydrophobic domain and an ionizable group [82]. However, some surfactants adversely affect the structures of PPs. The increase in lipophilicity is assessed by comparing the drug’s octanol or butanol/water partition coefficient, known as log(p), and the HIP complex. Incorporating PPs into HIP can impart sufficient lipophilicity and effective integration into lipid-based preparations [79]. These complexes may dissociate in response to changes in pH levels or salt competition in the GI environment. Consequently, hydrophobic ion-paired drugs are protected from the harsh environment of the GI tract, thus showing relatively higher permeability through the mucus layer and intestinal epithelium.

### 3.2. Mucus-Penetrating and Mucoadhesive Systems

The mucus lining along the intestinal membrane of the GI tract acts as a significant barrier to the absorption of PP therapeutics. However, mucus can represent both a benefit and a challenge when designing oral delivery systems for PPs. There are two opposing approaches for improving the efficacy of oral-based delivery systems: mucus-penetrating and mucoadhesive systems. Mucus-penetrating systems can rapidly pass through the undisturbed mucus layer to reach the intestinal epithelium for absorption. In contrast, mucoadhesive systems can prolong the drug residence time for absorption at the intestinal tract by avoiding clearance.

#### 3.2.1. Mucus-Penetrating Systems

Mucolytic agents, also known as mucus-penetrating agents, enhance drug permeation through the mucus barrier and improve the oral bioavailability of PPs (Figure 2) [83]. Initially used to disrupt the mucus barrier, mucolytic agents cleave the cross-links of disulfide bonds in secretory mucin, breaking its intermolecular network structure [83]. Mucolytic agents such as dithiothreitol, N-acetylcysteine, bromelain, and papain have been used in experimental animal models to assess the impact of the mucus layer on drug absorption in the GI tract [84]. These mucolytics aid in the attachment of particles to intestinal cells by removing the mucus layer covering the epithelium [51]. However, excessive removal of the mucus barrier can damage the intestinal epithelium by exposing it to proteolytic enzymes and digestive acids. Therefore, it is essential to use particles with specific surface charge properties for effective mucus penetration, as nanoparticles with positive and negative surface charges impede permeation.

Recent technological advancements have demonstrated that coating nanoparticles with PEG can make them hydrophilic, net-neutral, and densely surface-charged, resembling viruses [85]. PEG coating of nanoparticles reduces mucin adsorption, thereby enhancing the diffusion of particles through the mucus layer. Moreover, the geometry of the nanoparticle can significantly impact mucus-penetrating ability through micromotion. For instance, nanorods can move through mucus more quickly through rotation, allowing them to penetrate deeper into the mucus layer and remain in the GI tract for a longer duration.

#### 3.2.2. Mucoadhesive Systems

Various polymers with mucoadhesive properties have been used to develop nanocarrier systems to enhance the systemic absorption of orally administered PPs (Figure 2). These systems extend particle retention in the GI tract compared to non-adhesive systems. Nanocarriers often show non-specific mucoadhesion to the intestinal mucus. The mucoadhesive properties of polymers are governed by hydrophobicity, surface charge, and underlying chemical properties [86]. The interactions of negatively charged mucus with positively charged particles provide strong mucoadhesive properties. Chitosan, derived from chitin, is a biodegradable and biocompatible material often used in mucoadhesive systems [35]. More recently, N-trimethyl chitosan (TMC) has been used to engineer or coat nanoparticles [87]. This improves drug absorption through electrostatic interactions with mucins, as modified chitosan carries a positive charge that enhances their interaction with intestinal mucus through electrostatic forces. Van der Waals forces, hydrogen bonds, and hydrophobic interactions also play significant roles in these interactions [86].

Similarly, the thiolation of polymers by forming disulfide bonds with mucus glycoproteins represents another strategy for enhancing mucoadhesive properties. For example, N-trimethyl chitosan (TMC) nanoparticles modified with cysteine have shown increased insulin transport compared to their non-modified counterparts [88]. Likewise, molecules such as lectins adhere directly to epithelial cells instead of the mucus layer, unlike mucoadhesive polymers. They recognize receptor-like structures on cell membranes, allowing them to bind directly to the epithelium, thus potentially representing the next generation of bioadhesives [89,90]. Lectin-modified nanoparticles can extend residence time by binding to the intestinal epithelium and triggering active transport through receptor-mediated uptake. They are often used to target M cells to enhance the transport of large molecules [35,91].

### 3.3. Absorption Enhancement

#### 3.3.1. Prodrugs

To improve stability, solubility, or permeability, the prodrug strategy is the most common approach for modulating a drug’s physicochemical properties via chemical derivatization. Prodrug molecules overcome barriers and convert to their active form via degradation at the desired site of action [92]. These modifications include esterification, bio-reversible cyclization, and lipidation. Esterification enhances lipophilicity and intestinal permeability. Reversible cyclization of the peptide backbone strengthens intramolecular hydrogen bonding and minimizes interactions with water, making it a promising prodrug strategy for peptides [93]. Lipidation represents another promising approach for creating PP prodrugs to enhance hydrophobicity and intestinal permeability [35]. It may reduce a peptide’s biological activity; however, reversible lipidation techniques have already addressed this issue. This approach involves conjugating fatty acids with polypeptides in an aqueous solution, allowing the original active polypeptides to be regenerated after oral absorption [94]. Similarly, the prodrug design can achieve site-specific delivery by combining lipid rafts with a targeting moiety, thus facilitating rapid transport across the cell membrane. This technology may serve as an alternative method for improving the absorption of PPs.

While prodrug strategies offer significant advantages such as improved stability, solubility, and bioavailability, they currently show limited application in terms of modifying peptides. Chemical modification of PPs can be challenging, owing to their complexity and conformational instability during chemical reactions [35]. Furthermore, prodrugs with high lipid solubilities may bind to plasma proteins, decreasing the concentration of free drugs in the bloodstream and potentially interfering with specific receptor binding. Overall, prodrug strategies are valuable for improving the oral delivery of PPs, as these enhance their physicochemical properties and facilitate absorption. However, it is essential to carefully consider their limitations and potential drawbacks in order to optimize their usage in pharmaceutical formulations.

#### 3.3.2. Absorption Enhancers

Absorption enhancers represent a diverse group of chemical agents that improve drug absorption, particularly for PP therapeutics, by increasing their permeability through the intestinal epithelium [95]. These agents are essential in oral formulations where large molecules encounter barriers that hinder their efficient passage across the intestinal wall into the systemic circulation. They enhance drug transport through various mechanisms, including altering the structural integrity of the epithelial barrier by changing membrane fluidity and reducing mucus viscosity, which aids the passage of drugs through cells via the transcellular pathway because of increased membrane permeability or via the paracellular pathway by temporarily opening up the tight junctions between the epithelial cells. As most methods rely on opening these tight junctions to facilitate paracellular or targeted transcellular transport, they also inevitably damage these junctions in the process. However, these tight junctions account for a relatively small proportion of the intestine’s endothelial surface. Several absorption enhancers, including surfactants, fatty acids, chelators, glycerides, bile salts, salicylates, aromatic alcohols, ionic liquids, enterotoxin peptide derivatives, chitosan, and cell-penetrating peptides (CPPs) have been reported to promote transport across the intestinal epithelial cell barrier. Each absorption enhancer offers unique properties, distinct mechanisms of action, clinical applications, and limitations.

Surface active agents, also known as surfactants, are often used to improve the absorption of oral PPs across the GI tract. These amphipathic molecules are classified as anionic, cationic, nonionic, or zwitterionic. Particularly, non-ionic surfactants are generally used as excipients owing to their low toxicity and low reactivity with other ionic species [10]. They enhance permeation by integrating into the cell membrane, disrupting the lipid bilayer, and increasing membrane fluidity and transcellular transport [10]. Moreover, they have been shown to prevent the formation of protein aggregates while also inhibiting key intestinal enzymes such as α chymotrypsin [96]. Various surfactants, such as sodium dodecyl sulfate, sodium taurodihydrofusidate, and polysorbates, are commonly used [35]. Recently, medium- to long-chain fatty acid surfactants, such as capric and caprylic acids, have been used for the oral delivery of PPs, typically in the form of their sodium salts: sodium caprate and sodium caprylate. Sodium caprate enhances drug absorption by temporarily opening tight junctions through divalent cation chelation and facilitating transcellular transport via membrane disruption, efflux inhibition, and non-covalent protein interactions. These medium-chain fatty acids have been combined with other lipid excipients to enhance PP absorption. For instance, Merrion Pharmaceuticals’ gastrointestinal permeation enhancement technology (GIPET), licensed by Novo Nordisk, incorporates medium-chain fatty acids (capric and caprylic acids) and their derivatives, along with microemulsion systems based on medium-chain fatty acid glycerides. It is available in enteric-coated tablets or capsules designed for the oral administration of insulin, GLP-1 analogs, and hormones [97,98,99].

Similarly, Transient Permeability Enhancer (TPE^®^), developed by Chiasma Pharmaceuticals (Needham, MA, USA) comprises a proprietary combination of excipients, including sodium caprylate, which creates a lipophilic suspension of hydrophilic particles in a hydrophobic medium. This technology has been used in the oral delivery of octreotide (MYCAPSSA™) [100]. Recently, various benzoyl and salicyloyl derivatives of caprylic acid, butanoic acid, and capric acid, as well as their salts, such as N-(8-[2-hydroxybenzoyl]amino) caprylic acid, also known as salcaprozate sodium (SNAC), N-(5-chlorosalicyloyl)-8-aminocaprylic acid (5-CNAC), 4-([4-chloro-2-hydroxybenzoyl]amino) butanoic acid (4-CNAB), and N-(10-[2-hydroxybenzoyl] amino) decanoic acid (SNAD) are used as absorption enhancers for the oral delivery of PPs [10]. The non-covalent binding to the target PPs enhances hydrophobicity and prevents peptidase degradation at low pH. The complex dissociates in the intestine (pH > 7), facilitating transcellular transport. In 2019, Novo Nordisk formulated the first oral GLP-1, semaglutide (Rybelsus^®^) tablet for the treatment of T2DM using sodium N-[8-(2-hydroxybenzoyl) amino] caprylate (SNAC) as an absorption enhancer using Eligen^®^ technology, developed by Emisphere [101]. The utilized Eligen^®^ technology provides protection against gastric enzymes and improved lipophilicity to facilitate passive permeation through the intestinal epithelium. Furthermore, clinical studies have not indicated that SNAC significantly disrupts membrane integrity, alters membrane fluidity, or causes toxicity [102].

Chelating agents like ethylenediaminetetraacetic acid (EDTA), diethylenetriaminepentaacetic acid (DTPA), and ethylene glycol tetraacetic acid (EGTA) enhance paracellular absorption by binding calcium ions, which are crucial for tight junction integrity [10,35]. As EDTA is thought to increase paracellular transport by depleting extracellular Ca^2+^, it results in the disruption of epithelial barrier function and higher permeability [29,103]. Like EDTA, DTPA also inhibits intestinal proteases and disruption of tight junctions by nonspecifically chelating divalent metal ions (Ca^2+^, Mg^2+^, Zn^2+^). In addition to paracellular transport, EGTA shows stronger Ca^2+^ affinity. However, using chelators alone as enzyme inhibitors is impractical. EDTA conjugated to chitosan does not inhibit calcium-dependent proteases (trypsin, chymotrypsin, elastase) but effectively inhibits zinc-dependent proteases (carboxypeptidase A, aminopeptidase N) [10]. However, incorporating chelators into oral formulations presents in vivo challenges, such as maintaining effective concentrations without excessive dilution, avoiding cytotoxicity, and preventing significant reductions in trace element levels [104].

Similarly, zwitterionic compounds have been utilized in various applications for oral PP delivery. For instance, lauroylcarnitine and palmitoylcarnitine are two zwitterionic excipients used in the Peptelligence^®^ technology developed by Enteris BioPharma. These small molecules function as permeation enhancers by facilitating paracellular transport through tight junctions while also improving the solubility of the peptide cargo [105]. Another zwitterionic compound, palmityl dimethyl ammonio propane sulfonate (PPS, also known as 3- [N, N dimethyl (3-palmitoylaminopropyl) ammonio] propane sulfonate), features a quaternary ammonium group and a sulfate group. This molecule has shown effective intracellular delivery in vitro and facilitates the oral delivery of protein compounds such as salmon calcitonin in vivo [106].

Aromatic alcohols like propyl gallate, butylated hydroxytoluene, butylated hydroxyanisole, and their derivatives represent another class of small molecules that act as permeation enhancers and solubilizers to improve the transcellular transport of orally delivered PPs [107]. These antioxidants are widely used in both the pharmaceutical and food industries and are classified as GRAS excipients at their administered doses. However, chronic exposure to high levels of these compounds poses a carcinogenic risk. In a preclinical setting, Axcess™ delivery technology (Diabetology Ltd., Saint Helier, UK) has incorporated aromatic alcohols into several oral peptides, including Capsulin™ OAD (oral anti-diabetic for T1DM, phase IIb), Capsulin™ IR (insulin replacement for T1DM, phase II), Combulin (for T2DM), oral GLP-1 (for T2DM), and a combination of oral GLP-1 with insulin [108].

Likewise, bile salts serve as permeation enhancers by increasing drug absorption across biological barriers. These amphipathic biosurfactants enhance paracellular transport by loosening tight junctions, improve drug stability against enzymatic degradation, and fluidize intestinal cell membranes [109]. Several bile salts (sodium deoxycholate, sodium taurocholate, sodium glycodeoxycholate, and sodium taurodihydrofusidate) have been used to improve drug permeation across the intestine [110]. Nonetheless, their use in clinical settings is restricted due to potential cytotoxic effects such as irreversible membrane damage, irritation, and hemolysis.

Ionic liquids are another class of absorption enhancers for the oral delivery of PPs because of their unique solvating and permeation-enhancing properties. These liquids are made up of loosely coordinated anions and cations, including various cations (such as quaternary ammonium, imidazolium, pyrrolidinium, pyridinium, cholinium, and guanidinium) that have been used together with different anions (such as carboxylate, alkyl sulfate, dicyanamide, and bistriflimide) [10]. For instance, treatment with insulin in a choline and geranate (CAGE) ionic liquid showed a significant reduction in blood glucose levels when administered via oral gavage [111]. This ionic liquid possesses mucolytic activity resulting from decreased mucus viscosity, inhibits intestinal enzymes such as trypsin, and directly enhances permeation across the epithelial lining with minimal toxicity.

Toxins produced by bacteria and multicellular organisms have been used to develop permeation enhancers derived from specific purified toxin peptides. For instance, enterotoxin peptide derivatives like cholix toxin and zona occludens toxin (Zot), derived from the Gram-negative bacterium *Vibrio cholerae*, can reversibly increase paracellular permeability by activating intracellular signaling pathways that modulate actin polymerization [112] Similarly, other enterotoxin peptides and their derivatives include the *Clostridium perfringens* enterotoxin peptide and melittin facilitates the transcellular transport of proteins [113,114]. However, these peptides and their derivatives require additional protective measures to prevent them from being subjected to proteolytic degradation. Natural and synthetic, positively charged (cationic) polymers such as chitosan, chitosan derivatives, and cell-penetrating peptides (CPPs) are also reported as penetration enhancers for oral PPs. Despite their advantages, various absorption enhancers have potential drawbacks, including systemic toxicity and damage to the intestinal mucosa. Their prolonged or excessive use can disrupt membrane integrity, allowing harmful substances to be absorbed. Therefore, the safety profiles of absorption enhancers warrant further evaluation to mitigate the risks associated with their long-term use.

#### 3.3.3. Site-Specific Delivery

The absorption of PPs in the GI tract varies owing to differences in pH levels and the distributions of proteolytic enzymes. Moreover, the variable distribution of active and efflux transporters impacts the systemic absorption of oral PPs (Figure 2). A number of studies have been focused on identifying the optimal sites within the GI tract for absorbing PPs. The lower protease activity and higher pH in the colon make it an ideal site for PP absorption compared to the stomach and small intestine [115,116]. Various strategies can be used to ensure the intact delivery of oral PPs to the colon. One approach is to design PP prodrugs that remain stable in other regions of the GI tract but are converted to the active form, specifically in the colon [116]. This prodrug conversion can be facilitated by the microflora in the colon, which produce reductive enzymes capable of cleaving specific bonds (e.g., azo bonds) that link the prodrug to the active peptide [116]. Enzyme-controlled release mechanisms that exploit the enzymatic activity of the colon microflora are considered more reliable for delivering PPs to the colon. These microflora enzymes can activate various polymeric carrier systems to protect and release PP therapeutics at the optimal absorption site. Similarly, pH-sensitive delivery systems can release PPs in response to different pH microenvironments. However, these systems can also be affected by foods or pathological conditions that change the pH of the GI tract, potentially compromising their effectiveness.

#### 3.3.4. Active Targeting

A promising approach to enhance the oral absorption of PPs involves improving active transport by targeting receptors, transporters, and specialized cells within the intestinal epithelium [117]. Intestinal cells express a variety of transporters and receptors that interact with specific ligands such as vitamins and hormones. By utilizing these interactions, surface-functionalized nanocarriers with specific ligands can improve targeting to particular cell populations, facilitating more efficient absorption (Figure 2) [118]. Active targeting involves decorating nanocarriers with specific ligands to enhance interactions with intestinal epithelial cells and promote increased transport.

Various nanocarriers are often modified with vitamins such as folic acid (i.e., B9), biotin (i.e., B7), or thiamine (i.e., B1) to mimic their natural absorption pathways in enterocytes [35]. Folic acid is commonly used for this purpose and is absorbed by enterocytes via receptor-mediated endocytosis [119]. Its biocompatibility and strong affinity for folic acid receptors on enterocytes make it particularly attractive as a targeting ligand for oral nanocarriers. While this approach offers improved targeted absorption and enhances the likelihood of PPs reaching the systemic circulation, it also has potential drawbacks. These include the complexity of the nanocarrier design and the need to carefully balance biocompatibility with specificity in order to minimize off-target effects or limited bioavailability.

### 3.4. Lymphatic Transport

The gut lymphatic system, part of the broader circulatory system, represents an intricate drainage network that significantly impacts the oral absorption of PPs [120,121]. It regulates tissue pressure by draining fluids and proteins directly into the systemic circulation, thus bypassing the hepatic first-pass metabolism [122]. Lymphatic vessels in the intestines are specialized for absorbing dietary fats such as long-chain fatty acids, triglycerides, cholesterol, and other similar nutrients. Chylomicrons, the lowest-density lipoproteins, primarily comprise triglycerides and act as carriers in this transport system [122,123]. After passing through the intestinal epithelium, small hydrophilic drug molecules or macromolecules of <10 nm size (or 16–20 kDa for proteins) are mainly transported into the blood capillaries [35]. In contrast, highly lipophilic drugs can be incorporated into chylomicrons with lipoproteins and transported via the lymphatic system [124]. However, particles > 100 nm in size face difficulties in lymphatic transport because of limited diffusion and convection through the interstitium [125]. Lymphatic transport occurs in the intestinal lumen through lymphoid (e.g., Peyer’s patches) and non-lymphoid (e.g., villous) tissues. The transport through non-lymphoid tissues is influenced by multiple factors such as the lipid pathway, vehicle effects, sieving mechanisms of blood vessels, and the site of application.

The proximal small intestine is particularly effective for lymphatic transport; rectal administration has also shown potential for this pathway [35]. M cells in Peyer’s patches rapidly take up particles from the intestine via phagocytosis and transcytosis. Lipid-based formulations show promise in enhancing lymphatic transport by mimicking dietary fat absorption [35]. Particularly, unsaturated long-chain fatty acids can enhance the synthesis of chylomicrons and the lymphatic transport of hydrophobic drugs [62]. Excipients such as phospholipids, Tween 80, or TPGS can further enhance lymphatic transport [126]. Lipidation of peptides via chemical modification with fatty acids can represent a necessary approach to increase lymphatic transport by improving interactions with chylomicrons, and it has been applied in the oral delivery of several peptides [77]. Chylomicron uptake is the primary path by which drugs enter the lymphatic circulation [35]; however, they can also enter through gut-associated lymphoid tissue (GALT) via M cells. The delivery of PPs via the M cell pathway is limited because GALT makes up > 10% of the intestinal epithelial surface. After transcytosis, particles captured by M cells may end up in dome traps, potentially inhibiting the entry of PP therapeutics into the systemic circulation via lymphatic vessels, which can also limit the extent of PP absorption [35,127].

Moreover, lymphatic flow through the intestinal system is ~500 times slower than blood flow through the intestinal capillaries and portal vein [121]. This slower flow rate results in limited systemic absorption, reducing the overall bioavailability of oral PPs that target absorption through the lymphatic system. Overall, using the lymphatic route offers an alternative pathway for improving the oral delivery of PPs; however, limitations such as slower lymphatic flow and limited available surface area for absorption also pose certain unique challenges. Advances in lipid-based formulations and M cell-targeted delivery show promising avenues but must be carefully weighed against these inherent constraints.

**Table 1:** Strategies for enhancement of the oral absorption of PPs.

Strategy	Examples	Advantages	Disadvantages	Reference
pH Coating	Eudragit^®^systems, and hypromellose phthalate	Protects PP against enzymatic degradation in the stomach; pH-triggered systems provide controlled and targeted release in the intestines	Requires precise coating technology; potential delays in drug release, requires protease inhibitor and permeation enhancer in conjunction	[66]
pH Modulation	Citric acid	Protects against degradation in the stomach; targeted release in the intestines	Not effective across all pH ranges	[35,67]
Enzymatic inhibitors	Cholic acids, bestatin, aprotinin inhibiting trypsin, soybean trypsin inhibitor, camostat mesylate	Inactivates target enzymes by binding to their specific sites either reversibly or irreversibly	Toxicity at high concentrations and unpredictable reactions with PPs may affect the normal digestion of nutritive proteins	[68]
PEGylation	PEGylated insulin,	Prolongs circulation time; reduces immunogenicity; enhances stability	PEGylation can minimize cellular uptake; activity may trigger immune responses to PEG; conjunction technique could be complex	[18,72,128]
Cyclization	Cyclized somatostatin	Improves stability against enzymatic degradation; enhanced membrane permeability, selectivity for their targets, bioavailability	Complex synthesis process; potential reduction in bioactivity	[129,130]
Lipidation	Lipidated GLP-1	Enhances membrane permeability; improved oral bioavailability	Can affect peptide function; potential for increased lipid-induced toxicity	[131,132]
Mucopenetration	N-acetylcysteine, bromelain, papain	Enhances penetration through the mucus barrier	Risk of systemic absorption leading to side effects; complex formulation	[133]
Mucoadhesive	Chitosan, carbopol, polycarbophil, thiolated polymers, cellulose derivatives, pectin, xanthan gum	Increased PP concentration gradient at the epithelial barrier, prolongs retention time at the absorption site; enhanced membrane permeation	May cause local irritation; potential for unpredictable absorption due to mucus turnover at the absorption site	[134]
Prodrug	Dipeptide prodrugs, fatty acid prodrugs	Improved stability of PPs and bioavailability; targeted release	Difficult in prodrug design due to structural complexity; potential for toxic metabolites, and stability issues	[29]
Absorption Enhancer	Bile salts, fatty acids, surfactants, esters, cyclodextrin, dextran sulfate, crown ethers, EDTA, sodium caprate, and phosphatidyl choline	Improves intestinal permeability; enhanced bioavailability	Cause altered cell morphology, cause irritation or damage to the intestinal mucosa; transient effects, lack of specificity	[102,135]
Lymphatic Transport	Long-chain triglycerides based LDDSs	Avoid first-pass or presystemic metabolism	Limited to highly lipophilic drugs; complex formulation	[122,136]
Site-Specific Delivery	Folate-targeted nanoparticles, RGD vitamin B12, transferrin, invasins, and lectin	Lower systemic side effects; enhanced bioavailability	Requires specific targeting ligands; potential for off-target effects	[137,138]
Active Targeting	Peptide based-bioconjugates, Transferrin-Modified Carriers	Increases specificity to target tissues; improved therapeutic efficacy	Requires specific ligand-receptor interactions; complex manufacturing	[11,117,139]
Cell-penetrating peptides (CPPs)	TAT Peptide, Penetratin	Enhanced intramucosal delivery, and membrane permeability	Lack of cell specificity, endosomal entrapment, and potential immunogenicity	[140,141]

## 4. Clinical Applications of Oral PP Delivery

After the advent of the first recombinant insulin in 1981, many PPs have been investigated as new therapeutics in preclinical or clinical settings (Table 2). However, due to great challenges in oral delivery, only a few PPs are successfully administered via the oral route. Currently, marketed oral PP requires systemic absorption or retention in the GI tract to treat various diseases effectively (Table 3). Each delivery system utilizes single or combinational strategies to advance and accelerate the oral delivery of PPs. For instance, immunosuppressants like cyclosporine (Sandimmune^®^, Neoral^®^), a lipophilic cyclic polypeptide, are administered orally using SNEDDS approaches to prevent organ transplant rejection and treat autoimmune conditions such as psoriasis and rheumatoid arthritis [142]. Its hydrophobic nature and distinct molecular structure enhance intestinal absorption while protecting it from enzymatic degradation. The microemulsion preconcentrate-based peptide formulation enhances intestinal permeability and inhibits p-glycoprotein efflux and P450 metabolism.

**Figure 4:**
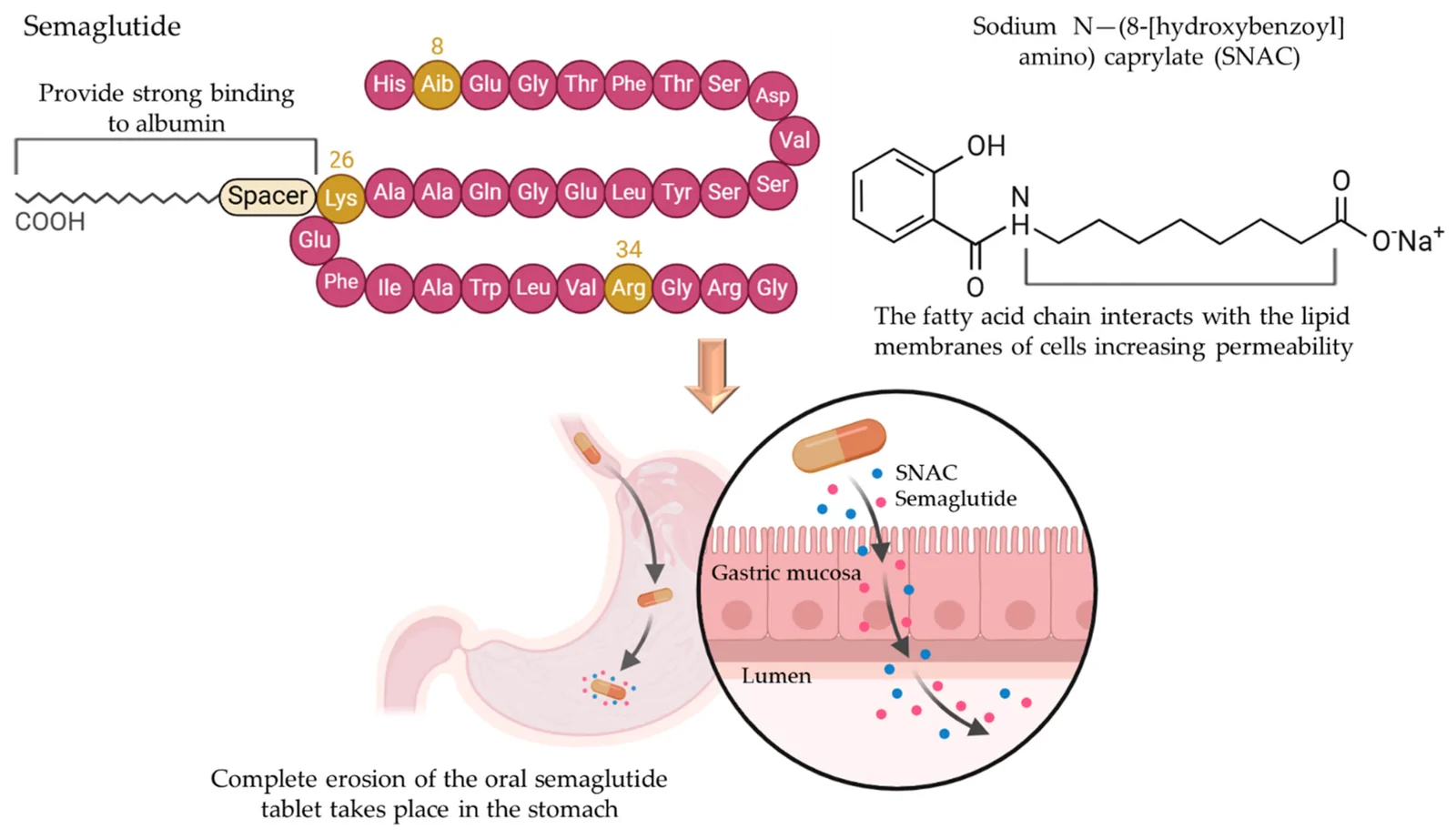
Delivery of oral semaglutide administration based on SNAC technology. SNAC enhances the oral delivery of semaglutide by raising the local gastric pH, protecting it from proteolytic degradation, and promoting monomerization. Additionally, it fluidizes lipid membranes, increasing their permeability and enabling efficient transcellular absorption of semaglutide into the systemic circulation. The figure was created using Biorender.com.

Similarly, Ferring Pharmaceuticals (Kastrup, Denmark) introduced desmopressin acetate (DDVAP^®^) as an oral tablet in 1995, followed by FDA approval of several generic versions [5]. Although chemical modifications, such as deamination of the first amino acid and substituting l-arginine with d-arginine at the eighth position, improved its stability, the oral bioavailability remains extremely low (~0.1%) due to the absence of permeation enhancer. Recently, Novo Nordisk developed Rybelsus^®^ (semaglutide), a GLP-1 analog approved by the FDA in September 2019, as an oral tablet based on Eligen^®^ SNAC technology. The developed formulation incorporates sodium N-(8-[2-hydroxybenzoyl]amino) caprylate (SNAC), a permeation enhancer to improve absorption (Figure 4) [101,143].

Furthermore, MYCAPSSA^®^ (octreotide), a synthetic somatostatin analog, was approved by the FDA in June 2020 using Transient Permeation Enhancer (TPE^®^) technology. The formulated enteric-coated capsule utilizes an oily suspension of octreotide with sodium caprylate as a permeation enhancer to temporarily open intestinal epithelial tight junctions, resulting in higher absorption via transcellular pathways. Despite a low oral bioavailability (~0.7%) requiring doses over 200 times higher than subcutaneous injections, clinical trials showed efficacy, making the twice-daily oral option preferable for many acromegaly patients over monthly depot injections.

In addition to systemic absorption, commercially available PP products such as colistin (Colomycin^®^), linaclotide (Linzess^®^), vancomycin (Vancocin^®^), and tyrothricin (Tyrozets^®^) act locally for different diseases, as mentioned in Table 3 [5]. As discussed in earlier sections, PP suffers from extreme conditions in the GI tract, including harsh pH, a richness in protease, a mucus layer, and a cellular barrier, which together result in limited systemic adsorption. To circumvent these barriers, various preclinical strategies have been proposed, including gastrointestinal permeation enhancement technology (GIPET^®^), Peptelligence™, ThioMatrix™, and POD^TM^ technology (Table 4). These strategies primarily focus on functional excipients, including intestinal absorption enhancers, protease inhibitors, mucoadhesive polymers, and nanocarriers. These technologies are still in the development or early clinical trial stages, but they provide new avenues for the clinical applications of oral PPs.

**Table 2:** Selected examples of oral PPs under clinical trial studies [144].

Product	PP	Company	NCAT Number	Status	Indication
ORMD-0801	Insulin	Oramed Ltd.	NCT01889667	Phase 3	T2DM
XW004	Ecnoglutide	Sciwind Biosciences	NCT05184322	Phase 1	T2DM, obesity
TransCon hGH	Somatropin	Ascendis Pharma	NCT01247675	Phase 2	Growth hormone deficiency
Somatropin	PEG-somatropin	Changchun GeneScience	NCT01342146	Phase 4	Growth hormone deficiency
RaniPill™ Capsule (RT-102)	Parathyroid hormone (1–34)	RANI Therapeutics	NCT05164614	Phase 1	Osteoporosis
RaniPill™ Capsule	Octreotide	RANI Therapeutics	NCT03798912	Phase 1	Growth hormone disorder
RaniPill™ Capsule (RT-111)	Ustekinumab	RANI Therapeutics	NCT05890118	Phase 1	Psoriasis
Ovarest™	Leuprolide	Enteris BioPharma Inc.	NCT02807363	Phase 2	Endometriosis

**Table 3:** Selected examples of commercially available systemically absorbed and locally delivered oral PPs.

Product	PP	Therapeutic Indications	Strategy
Sandimmune^®^/Neoral^®^	Cyclosporin A	Immunosuppression	SNEDDS, systemic delivery
Minirin^®^	Desmopressin acetate (DDVAP)	Cranial diabetes insipidus or nocturia associated with multiple sclerosis	Chemical modification, systemic delivery
Ceredist^®^/Ceredist OD^®^	Taltirelin hydrate	Spino cerebellar ataxia	Chemical modification to avoid enzymatic hydrolysis, systemic delivery
MYCAPSSA^®^	Octreotide	Growth hormone disorder	Enteric-coated capsules containing oil suspension and sodium caprylate as a permeation enhancer, systemic delivery
ColomycinC	Colistin	Intestinal infection (caused by sensitive Gram-negative organisms)	Acts locally to GI tract
Linzess^®^	Linaclotide	Irritable bowel syndrome, chronic idiopathic constipation	Acts locally on the luminal surface of the intestinal epithelium
Vancocin^®^	Vancomycin	Staphylococcus aureus and chlostridium difficile infection	Acts locally by inhibition of cell-wall biosynthesis.
Tyrozets^®^	Tyrothricin	Pharyngitis	Acts locally on the throat

**Table 4:** Key examples of recent technologies used in commercial oral PP delivery systems or clinical trials.

Technology	Strategy	Key Outcomes	Example	Reference
Transient Permeation Enhancer^®^ (TPE^®^ (Chiasma Pharmaceuticals))	Oily suspension with permeation enhancer sodium caprylate and polysorbate-80, open tight junctions and altering intestinal mucus thickness	Improved oral bioavailability	MYCAPSSA^®^ (octreotide)	[100]
Gastrointestinal Permeation Enhancement Technology (GIPET^®^) (Merrion Pharmaceuticals)	Incorporates medium-chain fatty acid derivatives coupled with salts and permeation enhancer (C10) to enhance hydrophobicity and epithelial tight junction opening.	Increased intestinal absorption, membrane fluidity, transcellular transport, and inhibited p-gp efflux	MER-101 (oral bisphosphonate for oncology, Phase-2), ACY-7 (Acyline, GnRHAntagonist, Phase-2)	[97,98,99]
Peptelligence™ Technology (Enteris Biopharma)	Enteric coating system containing pH-lowering agent (preferably citric acid), and permeation enhancer	Enhanced open tight junctions, facilitates paracellular transport and reduce protease activity and acid degradation.	TBRIA^TM^ (oral calcitonin, NDA), Ovarest^®^ (leuprolide oral tablet, Phase-2)	[145,146]
ThioMatrix™ Technology (ThioMatrix GmbH)	Incorporates thiolated mucoadhesive polymers (thiomers) to form covalent bonds with intestinal mucus glycoproteins for enhanced adhesion and retention.	Provides mucoadhesive, prolonged GI retention, permeation enhancement, and efflux pump inhibitory properties.	Hydrophilic macromolecules (Preclinical studies)	[147]
Oramed Technology (Oramed Pharmaceuticals)	Incorporates protease inhibitors and an absorption enhancer.	Protect PP from acid degradation and enhance intestinal permeation.	ORMD-0801 (Oral insulin, T2DM, Phase 3), ORMD-0801 (Oral insulin, NASH, Phase 2)	[148,149]
Protein oral delivery (POD^TM^) technology (Oramed Pharmaceuticals)	Incorporates protease inhibitors to protect therapeutic PP in the GI tract system.	Protect orally delivered PP from enzymatic activity, enhanced intestinal absorption.	ORMD-0901 (Oral GLP-1 capsule)	[150]

## 5. Advancement in Medical Device Technologies for Clinical Translation of Oral PP

Various innovative medical device technologies have been gaining traction to expand the clinical applications of oral PP. Currently, most device-based oral PP delivery has significantly improved the oral bioavailability of PPs by using strategies like patches, microneedles, and iontophoresis, which are adapted from previously established transdermal delivery [19]. As invasive needle-based devices aim to bypass the epithelium for higher bioavailability, this increases toxicological risks from mucosal perforation, which could worsen with muscular peristalsis and chronic use. However, microneedles that melt upon contact with the GI epithelium could help mitigate this risk. Inspired by transdermal microneedles, Rani Therapeutics (San Jose, CA, USA) developed the RaniPill™, a robotic pill for oral peptide delivery featuring sucrose-based microneedles activated by an osmotic, self-inflating balloon within an HPMC capsule. The pH-dependent coating dissolves in the upper GI tract, enabling microneedle insertion into the small intestinal epithelium. A preliminary animal model in pigs showed proof-of-concept with insulin-loaded devices manually placed in the jejunum to enhance delivery success [151].

**Figure 5:**
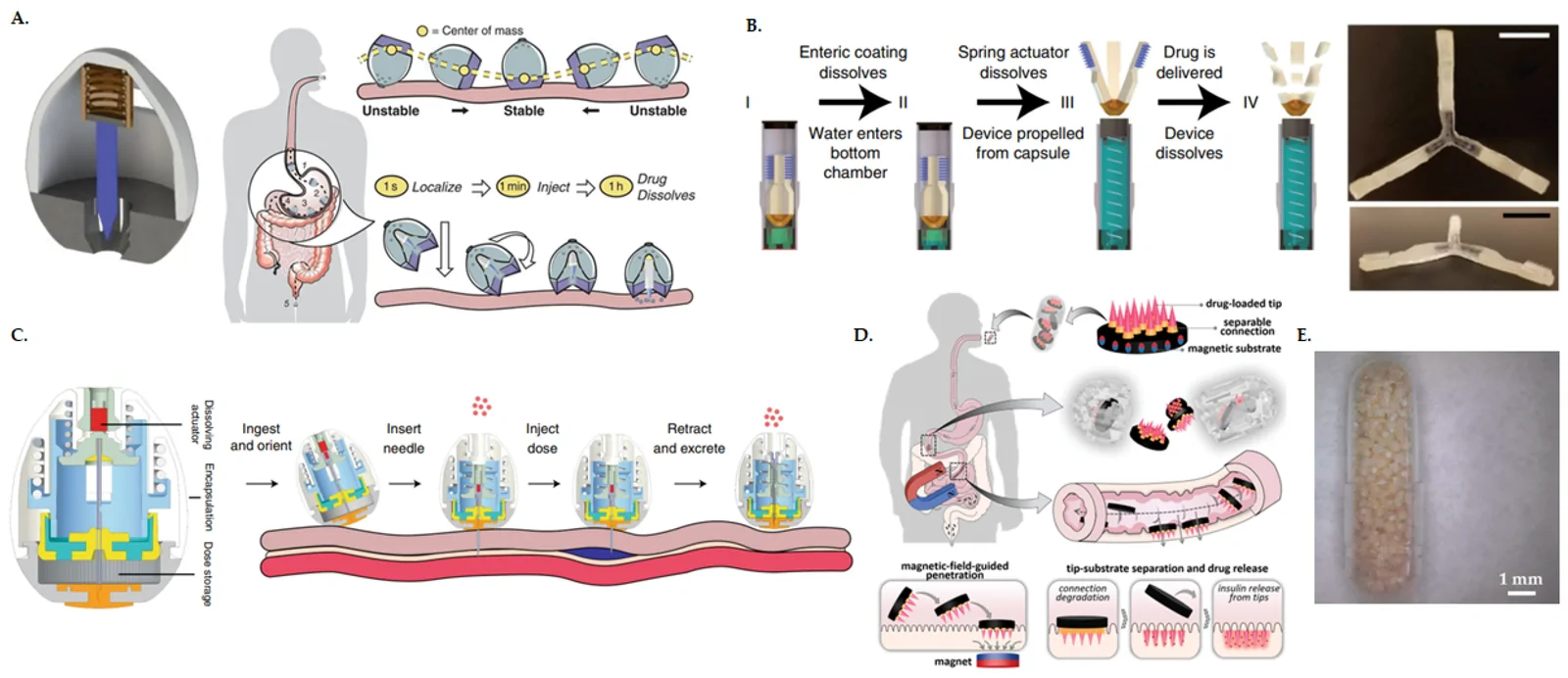
(**A**) Design of SOMA. The SOMA is designed to localize to the stomach lining, align its injection mechanism with the tissue wall, and deliver a drug payload through the mucosa. After the drug dissolves, the remainder of the device is expelled from the body. Self-orientation towards the desired upright position is provided by a high-curvature upper shell and a shifting of the center of mass. Once in its preferred orientation, the SOMA swiftly orients and stays stable in the stomach environment. Adapted from [152] with permission © 2019 American Association for the Advancement of Science. (**B**) Scheme of LUMI actuation, overhead (top), and side-view (bottom) images of an unfolded LUMI. Lumi devices were encapsulated in waterproof enteric capsules for ingestion. Upon reaching the small intestine, they actuated and unfolded, delivering drug-loaded microneedles in to the tissue wall. The microneedles patches and arms dissolved within a few hours, while the non-degradable components passed though the GI tract, and were eventually excreted. Adapted from [153] with permission © 2019 Springer Nature. (**C**) CAD design and device timeline of L-SOMA. Adapted from [155] with permission © 2021 Springer Nature. (**D**) Schematic illustrations of composition, release, drug delivery, and operational principle of the oral magneto-responsive microneedle robots (MMR). Adapted from [156] with permission © 2021 Wiley-VCH GmbH. (**E**) Micrograph showing a size 9 gelatin capsule containing coated micro-containers for delivering oral insulin [157].

Similarly, MIT and Novo Nordisk developed an oral self-orienting millimeter-scale applicator (SOMA) device to deliver insulin across the stomach wall (Figure 5A). The device can correctly self-orient to the gastric epithelium and actuates spring-loaded peptide-filled milliposts upon fluid ingress. This was the first study to show that gastric delivery to the systemic circulation can be achieved for peptides via physical disruption [152]. Recently, the SOMA team also developed a capsule-based injector that uses microneedles to deliver insulin through the small intestine, called the luminal unfolding microneedle injector (LUMI). These capsules comprise previously approved, osmotic-controlled release systems that use a pH-dependent methacrylate coating polymer (Figure 5B). Upon a rise in pH of >5.5, the capsule ruptures, and the spring is actuated, releasing entrapped, dissolvable microneedles from LUMIs. Each ejected three-folding arm has a microneedle array that penetrates the small intestinal mucosa with the force of actuation and releases insulin or other macromolecule drugs. Despite their functionality, SOMA and LUMI capsules faced key limitations, including low drug capacity (300–700 μg per pill), low absolute bioavailability (10% or less), and the requirement of enduring the degradative-enzyme-filled GI fluid before drug administration [153,154]. To solve these issues, they redesigned liquid-injecting SOMA (L-SOMA), enabling a higher drug-loading capacity (~4 mg) for liquid drugs like recombinant human insulin, GLP-1 analogs, adalimumab, and epinephrine directly into the gastric submucosa using dissolvable isomalt pellets to activate a needle- and plunger-based mechanism (Figure 5C). In a swine model, L-SOMA achieved plasma drug levels comparable to subcutaneous injections within 30 min and up to 80% bioavailability [155].

A recent study extended the design of ingestible devices by integrating them with external fields. Zhang et al. developed an innovative magnetically controlled microneedle robotic (MMR) device to deliver oral macromolecules (Figure 5D). This system, intended for enteric capsules, incorporates drug-loaded microneedle tips and a magnetic substrate that directs the tips to penetrate the intestinal wall using a magnetic guidance field. In diabetic minipigs, insulin-loaded MMRs penetrated 500 μm into the tissue, effectively normalizing blood glucose within 2 h and maintaining control even after glucose administration [156].

Patch systems and micro-containers are other alternative platforms for the oral delivery of PP. These designs enable unidirectional co-release and co-localization of peptides and permeation enhancers (PEs) at high concentration gradients near the epithelial wall, avoiding diluting and spreading the released payload in the GI lumen. Similarly, novel concepts, such as micro-containers, are also being developed specifically for oral delivery of PP. Jorgensen et al. designed Eudragit^®^-S100 coated micro-containers made of poly-ε-caprolactone, loaded with insulin and C10. In vitro studies show that proximity to the intestinal barrier improves insulin uptake, though in vivo challenges like micro-container retention and orientation in mucus hinder efficacy (Figure 5E) [157].

**Figure 6:**
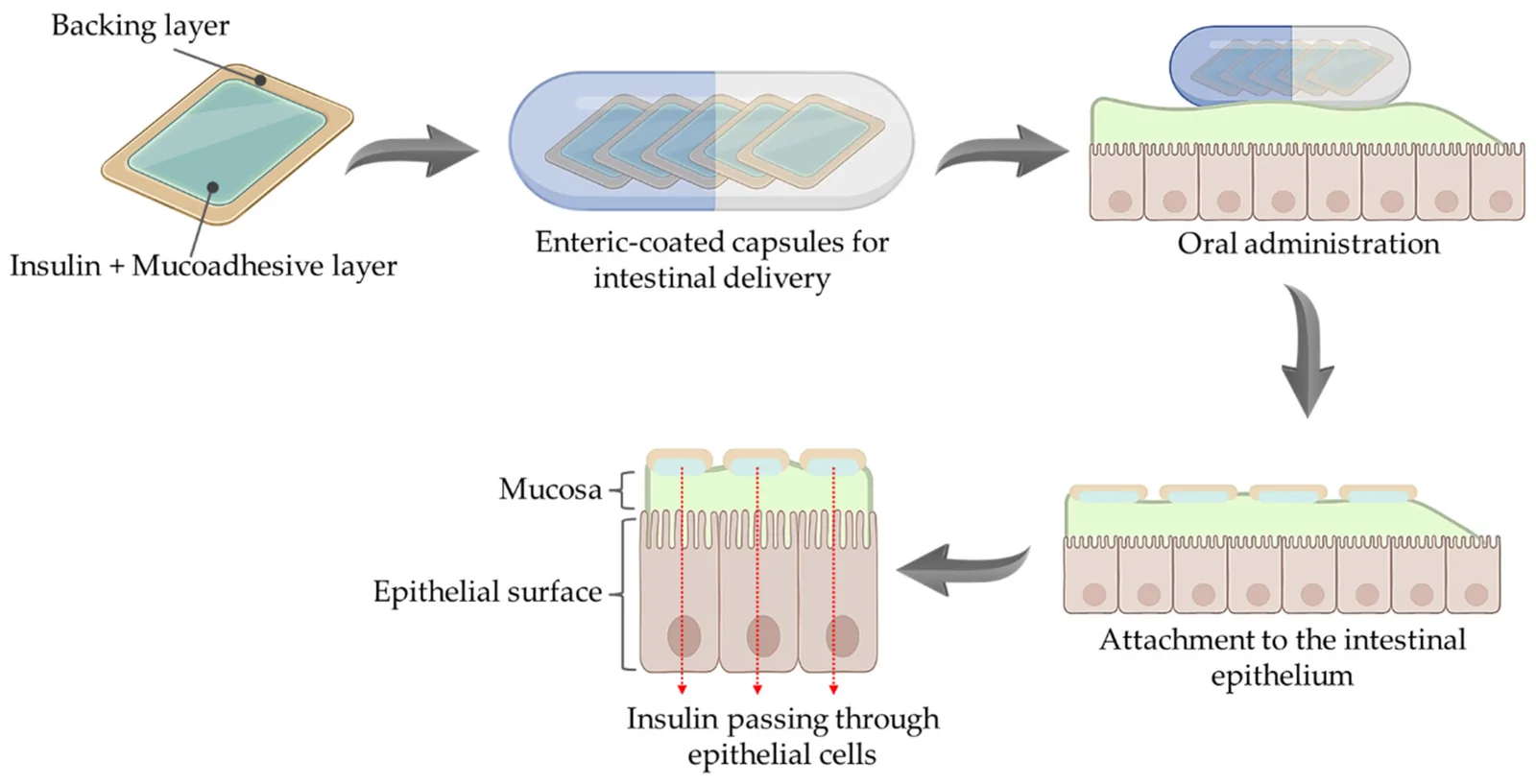
Delivery of oral PP loaded in mucoadhesive patches.

Likewise, non-invasive mucoadhesive intestinal micro patches are under investigation for oral delivery of PP [158,159,160]. For instance, Guptas et al. investigated oral PP delivery of insulin using Carbopol/Eudragit^®^ EPO, pectin, and CMC (carboxy methyl cellulose), dimethyl palmitoyl ammonia propanesulfonate (PPS) as the permeation enhancer, and citric acid as a peptidase inhibitor. These patches, with an ethyl cellulose backing, ensure unidirectional insulin release upon membrane attachment, demonstrating insulin’s pharmacodynamic effect in non-diabetic rats upon oral administration (Figure 6) [161]. Along these lines, an oral iontophoretic patch for insulin delivery was developed, demonstrating efficacy with electrically-activated insulin patches in rat models [161]. Despite these advancements, challenges persist for clinical translation, including limited drug-loading capacity, potential GI tract blockage or perforation risks, patient acceptance, and regulatory issues. Nonetheless, swift progress in material science and robotics may enhance the creation of patient-friendly ingestible devices for the oral delivery of PP, offering significant promise for clinical applications.

## 6. Future Directions and Conclusions

Over the past decade, notable strides have been made in the clinical translation of oral PPs. Despite significant advancements in nanotechnology and drug delivery systems, the prospect of successful clinical translation of oral PP remains bleak. While extensive research has yielded promising prototypes, only a handful of these systems advanced further in development, highlighting persistent challenges. These include poor stability of PP in the harsh GI environment and low permeability across intestinal barriers, resulting in poor bioavailability. The limited success in clinical translation underscores the urgency of innovative strategies.

The ongoing research primarily focuses on protecting against enzymatic degradation or permeation enhancement of PPs across the intestinal membrane. Recent progress in materials science and nanotechnology has introduced innovative platforms, such as lipid-based nanocarriers, polymeric nanoparticles, self-assembling peptides, stimuli-responsive hydrogels, and biomimetic systems. These advancements improve peptide stability and facilitate their passage through intestinal barriers. For instance, incorporating ligands specifically targeting intestinal receptors or exploiting endogenous transport pathways, such as those used by vitamins or bile acids, could greatly enhance the absorption of PP.

Likewise, a new strategy emphasizes the combination of cutting-edge nanotechnology with innovative medical devices. Microfabrication and nanofabrication techniques can create potential oral delivery devices, such as microneedle patches and micro-containers, which help PPs bypass physiological barriers to deliver them directly to mucosal surfaces. Similarly, innovative prototypes like smart-ingestible devices with external fields, sensors, and actuators achieve programmable PP release at specific sites. These systems may offer a controlled release profile, improve systemic absorption, and reduce enzymatic degradation, addressing multiple challenges simultaneously.

The synergy between delivery systems and patient-specific needs is an intriguing area for future exploration. Advancements in digital medicine, especially 3D bioprinting, enable personalized drug delivery systems tailored to individual physiology and disease state. These innovations could revolutionize treatments for chronic conditions like diabetes and inflammatory diseases, where long-term oral therapies are preferred. Nevertheless, maintaining a balance between stability, efficacy, and scalability presents a significant bottleneck. Additionally, regulatory aspects for intricate delivery systems need to be simplified to facilitate the transition from bench to bedside. By combining these innovative strategies with a comprehensive understanding of biological barriers and clinical requirements, researchers can pave the way for next-generation oral PP therapeutics with broader clinical applications and improved patient outcomes.
