# Correction: Baral, K.C.; Choi, K.Y. Barriers and Strategies for Oral Peptide and Protein Therapeutics Delivery: Update on Clinical Advances. *Pharmaceutics* 2025, *17*, 397

**DOI:** 10.3390/pharmaceutics18070864

**Published:** 2026-07-16

**Authors:** Kshitis Chandra Baral, Ki Young Choi

**Affiliations:** 1 Department of Marine Bio-Food Science, Gangneung-Wonju National University, Gangneung 25457, Republic of Korea; 2 NVience Inc., Seoul 04323, Republic of Korea

**Missing Citation**

In the original publication [1], ‘Plaza-Oliver, M.; Santander-Ortega, M.J.; Lozano, M.V. Current approaches in lipid-based nanocarriers for oral drug delivery. *Drug Deliv. Transl. Res.* **2021**, *11*, 471–497. https://doi.org/10.1007/s13346-021-00908-7’ was not cited. The citation has now been inserted in **3. Strategies for Improving Oral PP Absorption** and should read as follows:

**Figure 2.** Various strategies for efficient oral delivery of PPs (1) Stabilization of PPs in the harsh gastrointestinal environment against enzymes, salts, and microbiota. (2) Mucoadhesive system. (3) Enhancement of mucodiffusion through the mucus-penetrating agents. (4) Inhibition of drug efflux mechanism. (5) Active targeting. (5.1) Functionalized ligands that interact with specific cell populations (enterocytes or goblet cells). (5.2) Delivery system act as targeting ligands by themselves. (6) Enhancing lymphatic transport system. (6.1) Chylomicrons, including lipids and hydrophobic cargo molecules from the internalized nanocarriers, are generated within enterocytes and absorbed by the lymphatic system. (6.2) Lymphatic uptake can also be achieved via M cells. The figure was created using Biorender.com. Reproduced with permission from [2], Springer Nature, 2021.

With this correction, the order of some references has been adjusted accordingly. The authors state that the scientific conclusions are unaffected. This correction was approved by the Academic Editor. The original publication has also been updated.
